# Exposure to Environmental Antigens Induces the Development of Germinal Centers in Premature Neonates

**DOI:** 10.1080/1044667031000137665

**Published:** 2002-09

**Authors:** N. Wittenbrink, M. Zemlin, K. Bauer, C. Berek

**Affiliations:** 1 Deutsches Rheuma ForschungsZentrum Schumannstreet 21-22 Berlin 10117 Germany; 2 Universitäts Klinikum Benjamin Franklin Berlin Germany

## Abstract

The immune response of the neonate is poor and is dependent on passive immunity provided by maternal Ig. However, here we show that exposure of the neonate to environmental antigens induces a germinal center (GC) reaction. In the peripheral blood of premature infants one finds IgG class switched B cells expressing a selected V-gene repertoire. These data suggest that restrictions in the repertoire rather than immaturity of the immune system is responsible for the poor immune responses of the neonate.


*Developmental Immunology*, September 2002, Vol. 9 (3), pp. 177–179

### Exposure to Environmental Antigens Induces the Development of Germinal Centers in Premature Neonates*

N. WITTENBRINK[a], M. ZEMLIN[b,†], K. BAUER[b] and C. BEREK[a,‡]

[a]*Deutsches Rheuma ForschungsZentrum, Schumannstreet 21-22, Berlin 10117, Germany;* [b]*Universitäts Klinikum Benjamin, Franklin, Berlin, Germany*

The immune response of the neonate is poor and is dependent on passive immunity provided by maternal Ig. However, here we show that exposure of the neonate to environmental antigens induces a germinal center (GC) reaction. In the peripheral blood of premature infants one finds IgG class switched B cells expressing a selected V-gene repertoire. These data suggest that restrictions in the repertoire rather than immaturity of the immune system is responsible for the poor immune responses of the neonate.

*Keywords*: Class switch; Germinal center; Human neonate; V-gene repertoire

### INTRODUCTION

The V-gene repertoire is generated through the rearrangement of the Ig genes. The size of the repertoire is determined by the number of genes, their free combination and the diversity generated during joining of the different gene elements. In comparison to the adult V-gene repertoire, the fetal one is still restricted. As upstream $V_H$-gene elements become available for rearrangement only late in ontogeny, the fetal repertoire is characterized by an overrepresentation of certain gene elements (Schroeder *et al.*, 1995). Furthermore, the enzyme terminal transferase, which inserts nucleotides at the junction of the V(D)J-gene elements, becomes fully active only late in ontogeny. As the NDN-region forms the complementarity determining region III (CDRIII) of the H-chain and hence is the essential part of the antibody binding site, there are significant restrictions in the Ig gene repertoire of the neonate (Shiokawa *et al.*, 1999).

Furthermore, the immune system of the neonate seems to be unable to establish the micro environments necessary for the activation and differentiation of the antigen specific immune cells (Burgio *et al.*, 1990). The marginal zone develops only in the first year after birth, which may explain why the immune response of the neonate to bacterial infections is insufficient to protect the small infant. Furthermore, *in vitro* experiments showed that umbilical cord T lymphocytes fail to upregulate CD40 ligand upon activation (Durandy *et al.*, 1994). This immaturity of the antigen specific T cell may severely reduce the T cell help for the antigen activated B cell. It may thus prevent the development of GC.

A GC is the micro environment where the process of affinity maturation takes place (Berek *et al.*, 1991). During B cell proliferation in the GC hypermutation is activated. Variants are generated from a single B cell, which have receptors of different affinity for the antigen. Only those B cells with high affinity receptors are selected to differentiate into effector plasma or into memory cells. Here, we asked the question whether the immune system of the neonate is sufficiently developed to support a GC reaction.

At different time points after birth peripheral blood samples of preterm infants were analyzed. The Ig V-gene repertoire was determined using RT-PCR. The data suggest that exposure to environmental antigens induces Ig class switch. Furthermore, the V-gene repertoire is diversified by somatic mutation. Thus, exposure of the premature neonate (gestational age 25–29 weeks) to environmental antigens leads to the formation of GC.

### RESULTS

### Neonates Express a heterogeneous IgM Repertoire

The B cell V-gene repertoire in cord blood samples of term neonates (gestational week 35–42 weeks) was determined. The analysis of cDNA showed that B cells express a heterogeneous IgM V-gene repertoire. Since VDJ-

*Presented at the Proceedings of the 4th Germinal Center Conference, Groningen, The Netherlands, June 2002.
†Present address: Division of Developmental and Clinical Immunology, University of Albama, Birmingham AL, USA.
‡Corresponding author. Tel.: 49-30-28460-711. Fax: 49-30-28460-712. E-mail: berek@drfz.de

ISSN 1044-6672 print © 2002 Taylor & Francis Ltd
DOI: 10.1080/1044667031000137665

TABLE 1 $V_H4$-$J_H4$ rearrangements isolated from the peripheral blood of the premature neonate GA34

| A | B | C | V-gene | D-segment | CDR3 | Mutation |
|---|---|---|---|---|---|---|
| X | X | X | 4-04 | 2-15 | 14 | 3–6 |
| | | X | 4-04 | 2-02 | 11 | 4 |
| | X | | 4-04 | 7-27 | 14 | 0–2 |
| | X | X | 4-30.1 | 6-06 | 17 | 5 |
| | | X | 4-30.1 | 1-26 | 9 | 1 |
| X | X | X | 4-31 | 2-15 | 12 | 5 |
| X | X | | 4-39 | 4-17 | 13 | 6–8 |
| | X | X | 4-39 | 3-10 | 18 | 1 |
| | X | | 4-39 | 3-10 | 11 | 3 |
| | X | | 4-39 | 4-17 | 12 | 1–9 |
| | | X | 4-59 | 3-10 | 11 | 1 |
| | | X | 4-59 | 3-03 | 14 | 1 |
| | | X | 4-61 | 5-05 | 14 | 1 |
| X | | | 4-61 | 4-17 | 13 | 5 |

$V_H4$-$J_H4$ rearrangements isolated from three independent PCR reactions (A, B and C), $V_H4$ gene, D-segment, length of CDR3 and somatic diversity are indicated.

sequences with 5′ upstream $V_H$-gene elements were frequently seen, it would seem that at birth the Ig H-chain locus is completely open for rearrangement. The fetal overrepresentation of 3′-located $V_H$-genes, such as $V_H6$-01, was no longer observed. In comparison to adults, however, the V-gene repertoire of the term neonate is still restricted. The average length of the NDN-region (22 nucleotides) was significantly smaller than seen in adults (Zemlin *et al.*, 2001). Furthermore, it was difficult to detect IgG rearrangements. Only a few of the cord blood B cells have switched to the IgG class. In contrast to maternal IgG, there was no evidence for somatic mutations in these sequences.

### Exposure to Environmental Antigens Induces Class Switch

To see whether exposure of the premature neonate (gestational age 25–29 weeks) to environmental antigens induces class switch, blood samples were analyzed for the presence of IgG expressing B cells at different time points after birth. RNA was extracted from peripheral blood mononuclear cells and the IgG V-gene repertoire determined by RT-PCR. Table I shows results for the preterm infant GA34 (gestational age 27 weeks). At the age of 3 month, at the expected time of delivery, a diverse IgG repertoire developed. $V_H4$ rearrangements dominated the IgG response. From 3 independent RT-PCR reactions 13 different $V_H4$-$J_H4$ rearrangements were isolated (Table I). This broad spectrum of different IgG rearrangements demonstrated that the immune system of the premature neonate is able to respond to antigenic stimulus. Exposure to environmental antigens induced both B cell activation and Ig class switch.

### Evidence for GC Reaction

The analysis of the IgG V-region genes showed both, unmutated sequences and those with multiple somatic mutations (Table I). In addition, identical VDJ-rearrangements with different patterns of somatic mutations were isolated. These data suggest that exposure to environmental antigens induced GC formation in the premature neonate. During B cell proliferation in the GC nucleotide substitutions accumulated stepwise in the V-region genes. The analysis of the mutational pattern showed that somatic mutations are non-randomly distributed. The preferential accumulation of replacement mutations in the CDR of the $V_H$-genes suggested that selection for high affinity variants has taken place.

### MATERIAL AND METHODS

Cord blood of healthy neonates and peripheral blood of premature neonates at their expected date of delivery was analyzed. Data are shown for the premature neonate a42 (gestational age 27 weeks) (Bauer *et al.*, 2002). During the first 3 months of life the premature neonate GA34 developed four episodes of bacterial infection.

### RT-PCR

RNA was extracted from 200 μl of heparinised blood (QIAamp RNA Blood Mini kit) and RT-PCR performed. cDNA was transcribed using primers specific for the $C_H$ region of IgG and amplified with primers specific for the first framework of the different $V_H$-gene families. For the reamplification primers specific for $V_H4$ and $J_H4$ were used.

### $V_H$ Gene Analysis

$V_H$-region genes were sequenced using the ABI system (Perkin Elmer). To determine the $V_H$-gene diversity, sequences were compared with the putative germline gene using V-base (I. Tomlinson, Cambridge, UK).

*Acknowledgements*

We would like to thank I. Gläser and G. Steinhauser for technical assistance. The work was supported by the Deutsche Forschungs Gemeinschaft (grant to BA 1187). The Deutsche RheumaForschungszentrum is supported by the Berlin Senate for Research and Education.

***References***

Bauer, K., Zemlin, M., Hummel, M., Pfeiffer, S., Karstaedt, J., Steinhauser, G., Xiao, X., Versmold, H. and Berek, C. (2002) “Diversification of Ig heavy chain genes in human preterm neonates prematurely exposed to environmental antigens”, *J. Immunol.* **169**, 1349–1356.

Berek, C., Berger, A. and Apel, M. (1991) "Maturation of the immune response in germinal centers", *Cell* **67**, 1121–1130.

Burgio, G.R., Ugazio, A.G. and Notarangelo, L.D. (1990) "Immunology of the neonate", *Current Biology* **2**, 770–777.

Durandy, A., de Saint Basile, G., Lisowska-Grospierre, B., Gauchat, J.-F., Forveille, M., Kroczek, R.A., Bonnefoy, J.-Y. and Fischer, A. (1994) "Undetectable CD40 ligand expression on T cells and low responses to CD40 binding agonists in human newborns", *J. Immunol.* **154**, 1560–1568.

Schroeder, H.W., Mortari, F., Shiokawa, S., Kirkham, P.M., Elgavish, R.A. and Bertrand, F.E. (1995) "Developmental regulation of the human antibody repertoire", *Ann. NY Acad. Sci.* **764**, 242–262.

Shiokawa, S., Mortari, F., Lima, J.-O., *et al.*, (1999) "IgM heavy chain complementarità determining region 3 diversity is constrained by genetic and somatic mechanisms until two months of birth", *J. Immunol.* **162**, 6060–6070.

Zemlin, M., Bauer, K., Hummel, M., Pfeiffer, S., Devers, S., Zemlin, C., Stein, H. and Versmold, H.T. (2001) "The diversity of rearranged immunoglobulin heavy chain variable region genes in peripheral blood B cells of preterm infants is restricted by short third complementarity-determining regions but not by limited gene usage", *Blood* **97**, 1511–1513.

